# A strategy to formulate data-driven constitutive models from random multiaxial experiments

**DOI:** 10.1038/s41598-022-26051-y

**Published:** 2022-12-23

**Authors:** Burcu Tasdemir, Antonio Pellegrino, Vito Tagarielli

**Affiliations:** 1 Department of Engineering Science, University of Oxford, Oxford, UK; 2 Department of Aeronautics, Imperial College London, London, UK

**Keywords:** Aerospace engineering, Mechanical engineering

## Abstract

We present a test technique and an accompanying computational framework to obtain data-driven, surrogate constitutive models that capture the response of isotropic, elastic–plastic materials loaded in-plane stress by combined normal and shear stresses. The surrogate models are based on feed-forward neural networks (NNs) predicting the evolution of state variables over arbitrary increments of strain. The feasibility of the approach is assessed by conducting virtual experiments, i.e. Finite Element (FE) simulations of the response of a hollow, cylindrical, thin-walled test specimen to random histories of imposed axial displacement and rotation. In these simulations, the specimen’s material is modelled as an isotropic, rate-independent elastic–plastic solid obeying J2 plasticity with isotropic hardening. The virtual experiments allow assembling a training dataset for the surrogate models. The accuracy of two different surrogate models is evaluated by performing predictions of the response of the material to the application of random multiaxial strain histories. Both models are found to be effective and to have comparable accuracy.

## Introduction

Applications of machine learning have exponentially increased in number during the last decade and have spread to different fields including image recognition, web search, voice recognition, financial services, healthcare, sales, and more. Recent research also explored applications of machine learning to the mechanics of solids, with the main objective of reducing the computational cost of simulations involving complex material behaviour, by the introduction of surrogate constitutive models or data-driven microstructure-properties relations^1–3^. This exercise is particularly difficult in the presence of history-dependent responses^4^. Some authors have attempted using recurring NNs to capture non-linear, path-dependent constitutive responses of different types of solids^5^; considerable work has explored the response of heterogeneous solids, mainly two-phase composites, and used data-driven approaches to predict the effects of their microstructure on the mechanical behaviour (e.g.^6^). Some researchers have worked on data-driven models for elastic–plastic solids, collecting training datasets from simulations or measurements of proportional loading histories^7–9^, or from the results of structural tests^10^. We recently proposed a computational procedure to develop surrogate constitutive models for multiphase random composites^11^ with time- and path-dependent response, suitable for arbitrary multi-axial and non-proportional loading.

Several of the data-driven models described above were trained with numerical simulations of the response of Representative Volume Elements (RVEs) or of single FEs of known constitutive description. Similar approaches can in principle be developed using data obtained directly from measurements, to implement model-free simulations, as shown effectively by^10,12–16^. No previous research however has focused on developing data-driven constitutive models for elastic–plastic materials starting from data obtained from measurements and including non-monotonic and non-proportional loading; this is the objective of the present study.

Here we follow similar steps as in^11^ to obtain surrogate constitutive descriptions of the response of solid materials from measured data collected in lab-scale experiments. The objective is to develop numerical descriptions of the material behaviour which do not rely on any particular theory and are able to predict the material response subject to arbitrary loading, including complex non-monotonic and non-proportional deformation histories. To prove the feasibility of the approach, we conduct virtual tests by performing FE simulations of the response of a hollow, cylindrical, thin-walled test specimen to random histories of imposed non-proportional axial displacement and torsional rotation. The results of the simulations are processed in the same way as in actual experiments, obtaining time histories of the average direct and shear stresses and strains in the specimen’s gauge portion. Repeated virtual experiments are conducted by imposing multiple random deformation histories, such to adequately sample the space of the applied deformation. Such strain and stress histories are subdivided in small time increments, and the stresses and strains at the beginning and at the end of each increment are used as inputs and outputs, respectively, for the surrogate models. Additional history-dependent inputs are also included in the model. NNs are used to establish a correspondence between the inputs and the outputs, after appropriate training. The choice of obtaining the training dataset from virtual (rather than actual) experiments is driven by two reasons: (i) the fact that in virtual experiments the material’s response is well-defined and known in detail and (ii) the desire to perform a feasibility study for this approach, before embarking in an extensive experimental campaign.

The paper is structured as follows. In section “Design and analysis of the virtual experiments” we illustrate the design of the virtual test specimens, of the FE simulations of their response, and of the analysis of the simulation data; section “Implementation of the surrogate models” presents the strategies to create the training dataset and to implement the surrogate models; results are presented and discussed in section “Results and discussion”.

## Design and analysis of the virtual experiments

### Virtual test specimen

Performing an accurate and complete characterisation of the multiaxial stress–strain response of solids is challenging, costly and requires equipment such as triaxial test machines, which are unavailable to most materials engineers. However, applying a combination of axial strain and torsional rotation to a thin-walled axisymmetric material specimen (which is the strategy pursued in this study) is relatively inexpensive and it results in approximately uniform normal and shear stresses on the specimen’s gauge portion; this allows exploring the material’s response in plane stress.

**Figure 1:**
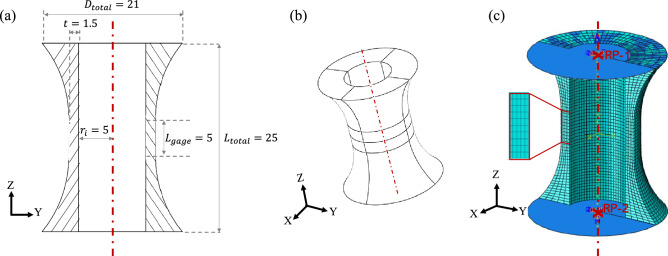
The geometry of the virtual specimen; (**a**) cross-sectional view; (**b**) 3-D view; (**c**) FE mesh and boundary conditions (all dimensions in mm).

The design of the test specimen was performed to minimise the variability of the normal and shear strains and stresses within the gauge portion, and to prevent compressive and torsional buckling and wrinkling during the (virtual or real) experiments. The geometry of the specimen, sketched in Fig. 1, was selected considering the existing literature on fatigue and cyclic plasticity testing^17–20^. The detailed sizing of the specimen was performed with the aid of FE simulations of its elastic–plastic response in pure tension, pure compression and pure torsion; the simulations served to assess and maximise the uniformity of the strains and stresses in the gauge portion; checks were performed to ensure that compressive and torsional elastic buckling did not occur, based on theoretical predictions for a thin-walled hollow cylinder (from^21^), with the conservative assumption of simply-supported boundary conditions at the ends of the cylinder. The final dimensions are indicated in Fig. 1.

### Finite element analyses

All simulations were conducted using the commercially available software Abaqus Standard^22^. The material of the virtual specimens was taken as isotropic linear elastic (with Young’s modulus of 100 GPa, Poisson’s ratio $\nu = 0.3$), with rate-independent J2 plasticity and isotropic hardening (with initial yield stress $\sigma_{Y} = 1\;{\text{GPa}}$ and constant hardening modulus $H = \partial \sigma_{Y} /\partial \hat{\varepsilon }_{pl} = 5\;{\text{GPa}}$, where $\hat{\varepsilon }_{pl}$ represents the von Mises equivalent plastic strain).

The geometry of the FE model coincided with that shown in Fig. 1, comprising the gauge portion and both specimen shoulders, but excluding the gripping portions of the specimen. The domain was meshed by solid, 8-noded hexahedral elements with reduced integration (type C3D8R in Abaqus). After a mesh convergence study, the gauge portion of the specimen was discretised by a structured mesh with elements of dimensions 0.3 and 0.5 mm in the radial and axial directions, respectively, and 80 elements along the circumferential direction, as shown in Fig. 1c.

Two analytical rigid surfaces were defined at the top and bottom of the specimen and connected to the nodes at the ends of the specimen via tie constraints. Reference points were introduced for both rigid surfaces; one of the reference points had all 6 degrees of freedom constrained to vanish; the other reference point had all degrees of freedom set to zero, with the exception of the translation along direction *y* and the rotation about the *y* axis. Although in principle they over-constrain the specimen’s ends compared to a real test, these boundary conditions were chosen for the simplicity of their implementation, after a preliminary study which showed that the presence of the additional constraints on the radial displacements of nodes at the specimen’s ends did not affect the stress versus strain histories recorded in the virtual tests. Axial and torsional loading was applied by prescribing appropriate translation and rotation histories using the “amplitude” tool of Abaqus; details of this will be given below.

The axial force and torque applied to the specimen during the virtual tests coincided with the reaction force and torque at the loading reference point, and were recorded as a function of time during the simulations. Knowledge of the specimen’s geometry allowed calculating the applied true stresses (axial stress $\sigma_{z}$ and shear stress $\tau$); the displacements of appropriate nodes on the surface of the specimen (within the gauge portion) were tracked and used to calculate the history of the normal strains in the axial and circumferential direction ($\varepsilon_{z}$ and $\varepsilon_{\theta }$, respectively) and of the shear strain $\varepsilon_{\theta z}$; we also checked that the radial strain $\varepsilon_{r}$ was approximately equal to $\varepsilon_{\theta }$, as expected for this isotropic material at small strains.

The results of the multiple virtual experiments conducted were in line with the expected response of the material, in terms of the elastic compliance, shape, magnitude and symmetries of the initial yield locus, as well as hardening characteristic. This reassures us that the design of the specimen was adequate to characterise the material’s response.

## Implementation of the surrogate models

### Generation of the training data set

We now illustrate the assembly of the training dataset for the surrogate constitutive models. The objective was to sample, as uniformly as possible, the input space for the surrogate models; more precisely, to sample the space of the inputs that can be controlled during the virtual tests, i.e. the average axial and shear strains $\varepsilon_{z}$ and $\varepsilon_{\theta z}$ (respectively) in the gauge portion of the specimen. A further requirement was that such strains were sufficiently small to avoid the possibility of damage (in real measurements) and of strain localisation and other instabilities, as well as strain-induced anisotropy (in both virtual and real measurements).

Random histories of $\varepsilon_{z}$ and $\varepsilon_{\theta z}$ were generated starting from an initial undeformed configuration; such histories comprised a random sequence of imposed deformation steps $\left( {\Delta \varepsilon_{z} ,\;\Delta \varepsilon_{\theta z} } \right)$, each comprised between a minimum and maximum value, and terminating when one of the imposed strain components reached a limiting value, taken in this study as 0.04 for the engineering values of both $\varepsilon_{z}$ and $\varepsilon_{\theta z}$. Mathematically:

1 $$\begin{aligned} \Delta \varepsilon_{z} & = {\text{sign}}\;\left( {0.5 - r} \right)\;\left[ {\Delta \varepsilon_{{\text{min}} } + r\;\left( {\Delta \varepsilon_{{\text{max}} } - \Delta \varepsilon_{{\text{min}} } } \right)} \right]; \\ \Delta \varepsilon_{\theta z} & = {\text{sign}}\;\left( {0.5 - r} \right)\;\left[ {\Delta \varepsilon_{{\theta z} {{\text{min}} }} + r\;\left( {\Delta \varepsilon_{{\theta z} {{\text{max}} }} - \Delta \varepsilon_{{\theta z} {{\text{min}} }} } \right)} \right]. \\ \end{aligned}$$

where *r* is a random number generated from a uniform distribution in the interval [0, 1]. In this study we choose $\Delta \varepsilon_{{z}{{\text{min}} }} = \Delta \varepsilon_{{\theta z}{{\text{min}}} } = 0.005$ and $\Delta \varepsilon_{z{\text{max}} } = \Delta \varepsilon_{{\theta z}{{\text{max}} }} = 0.01$, to avoid extremely small or extremely large deformation steps; this guarantees random stain histories that are not too long or too short (respectively), and it helps sampling wide ranges of imposed strain triaxiality. The term containing the sign() function in Eq. (1) applies a random sign to each of the strain increments. The histories of imposed axial displacement and torsional rotations were calculated assuming that the shoulder portions of the specimen were rigid.

An additional set of virtual tests was conducted to impose states of uniaxial stress (tension, compression and shear) on the specimen, with the objective of determining the elastic compliance matrix of the material **S**. This was defined as

2 $$\left( {\begin{array}{*{20}l} {\Delta \varepsilon_{z} } \\ {\Delta \varepsilon_{r} = \Delta \varepsilon_{\theta } } \\ {\Delta \varepsilon_{\theta z} } \\ \end{array} } \right) = \left[ {\begin{array}{*{20}l} {S_{11} } & {\quad S_{12} } & {\quad 0} \\ {S_{12} } & {\quad S_{22} } & {\quad 0} \\ 0 & {\quad 0} & {\quad S_{33} } \\ \end{array} } \right]\left( {\begin{array}{*{20}l} {\Delta \sigma_{z} } \\ 0 \\ {\Delta \tau } \\ \end{array} } \right) = \left( {\begin{array}{*{20}l} {S_{11} \Delta \sigma_{z} } \\ {S_{12} \Delta \sigma_{z} } \\ {S_{33} \Delta \tau } \\ \end{array} } \right).$$

The components of **S** were determined by a least square fit of Eq. (2) through the first 80 increments of each of these simulations, in which the response was purely elastic.

**Figure 2:**
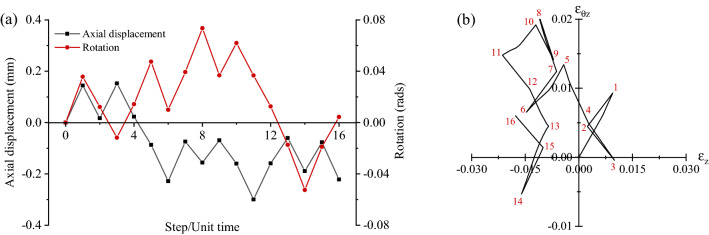
(**a**) Example of a 16-step history of imposed random displacement and rotation; (**b**) corresponding path in strain space.

**Figure 3:**
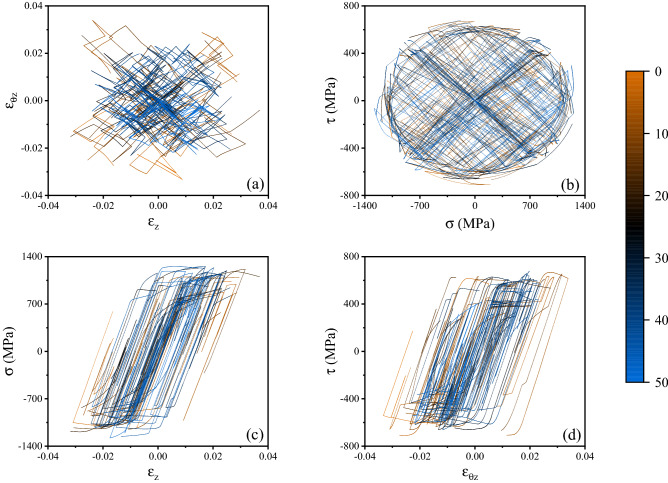
Visualisation of the training dataset (**a**) in strain space; (**b**) in stress space; (**c**) in axial stress versus strain space; (**d**) in shear stress versus strain space. Each of the fifty different loading paths is associated to a different colour according to the colour code displayed on the right.

Figure 2 illustrates a representative history of imposed random displacement/rotation and the corresponding path in the strain space. We note that each deformation step occurs over a time of 1. Fifty of such random strain histories were applied in the corresponding virtual experiments. The complete data set is shown in Fig. 3 in strain space $\left( {\varepsilon_{z} ,\;\varepsilon_{\theta z} } \right)$ and stress space $\left( {\sigma_{z} ,\;\tau } \right)$; the corresponding axial and shear stress versus strain histories $\sigma_{z} \;{\text{vs}}\;\varepsilon_{z}$ and $\tau \;{\text{vs}}\;\varepsilon_{\theta z}$ are also presented. The time increment for the FE solution was set to 0.01, such that each deformation step (of time duration 1) comprised 100 time increments. The 50 random strain histories comprised a total of 552 deformation steps and therefore 55,200 time increments. The number of random strain histories used to generate the training dataset was determined by analysing the sensitivity of the predictions to the size of the training dataset, as described in more detail in the Appendix (Supplementary material).

**Figure 4:**
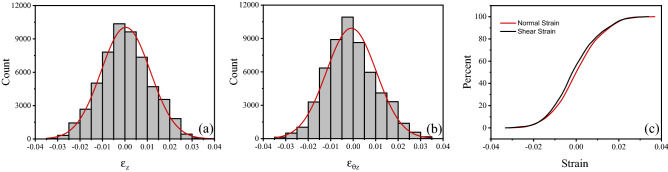
PDF and CDF of the applied axial and shear strains.

We note that the distribution of the data in true strain space (Fig. 3a) is not uniform in the square $\varepsilon_{z} ,\;\varepsilon_{\theta z} \in \left[ { - \,0.04,\;0.04} \right]$; data are distributed more densely in the proximity of the origin, as all random strain histories start at the undeformed state; the density of data is also low near the boundary of the domain, due to the fact that deformation steps with $\varepsilon_{z}$ or $\varepsilon_{\theta z}$ exceeding the maximum value of 0.04 were discarded and the corresponding random strain history was terminated. The probability density functions (PDF) and its cumulative counterparts (CDF) for $\varepsilon_{z}$ and $\varepsilon_{\theta z}$ are shown in Fig. 4. The uniformity of such distribution can be improved by creating additional random deformation histories and modifying the strategy (Eq. (1)), however the data in Fig. 3 was found adequate to give a good accuracy of the surrogate models.

### Separation of elastic and elastic–plastic increments

A further manipulation of the training dataset was performed in order to separate elastic from elastic–plastic increments. This was done by recording, for every increment, the change in external work $\Delta W$ and dissipated plastic energy $\Delta PE$, as obtained from the FE simulations. Each increment was then assigned a flag value $Y$ given by

3 $$Y = \left\{ {\begin{array}{*{20}l} 1 & {\quad \Delta PE/\left| {\Delta W} \right| \ge 0.01} \\ 0 & {\quad \Delta PE/\left| {\Delta W} \right| < 0.01} \\ \end{array} } \right.$$

with $Y = 1$ corresponding to an elastic–plastic increment and $Y = 0$ corresponding to an elastic increment. The choice of using energies to perform such separation was dictated by convenience; in real mechanical tests, where such energies are not normally known, such separation can be performed by computing the plastic strain components. We note that the threshold of 0.01 used in Eq. (3) was determined by trial and error in a preliminary study, by assessing its effects on the accuracy of the classification. We found that too high values of the threshold imposed on ΔPE/|ΔW| caused a set of the elastic–plastic increments to be incorrectly categorised as elastic. Conversely, too small a threshold resulted in a fraction of the elastic increments to be incorrectly classified as elastic–plastic. A range of values of such threshold resulted in a correct classification for all increments, and the value of 1% was chosen arbitrarily within such range.

**Figure 5:**
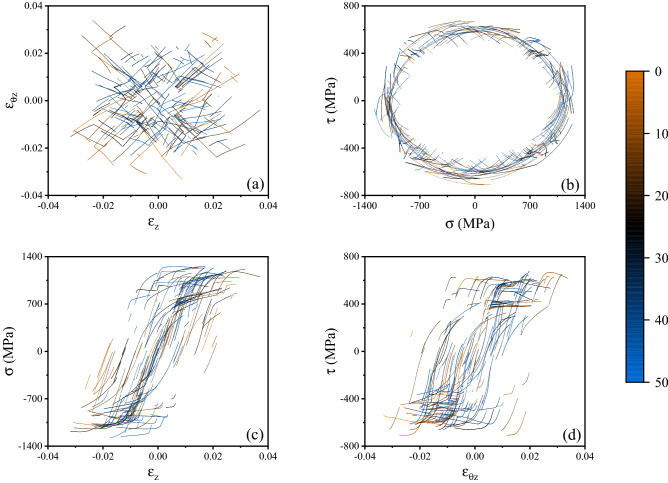
Visualisation of the plastic increments in the training dataset (**a**) in strain space; (**b**) in stress space; (**c**) in axial stress versus strain space; (**d**) in shear stress versus strain space. Each of the fifty different loading paths is associated to a different colour according to the colour code displayed on the right.

Figure 5 visualises the same dataset as in Fig. 3 after exclusion of the elastic increments with $Y = 0$; this comprises 16,500 elastic–plastic increments.

### Surrogate models

Feed-forward neural networks (NNs) were used here to construct surrogate constitutive models based on the data presented above. A summary of how NNs work can be found in^23^. The NNs were developed and trained in TensorFlow 2.0^24^. Our objective is to develop constitutive models that can be used in numerical simulations; these consist of computational frameworks receiving as input a tentative increment in the strain vector at a material point and returning the corresponding increment in the stress vector.

**Figure 6:**
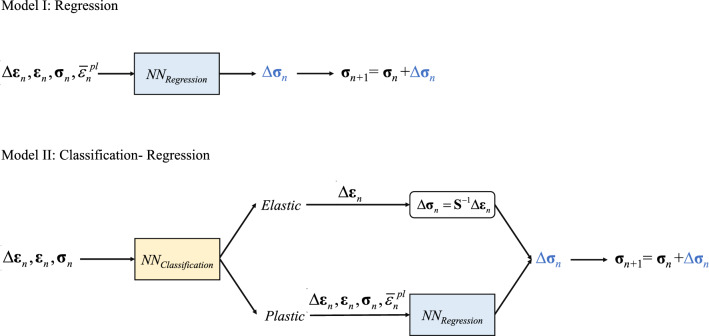
Schematic of the two surrogate models.

Two different surrogate models are presented in this study, and their architecture is summarised in Fig. 6. Model I performs a simple regression to update the stress state over each simulation increment. Model II first performs a classification step, to distinguish elastic from elastic–plastic increments; then, if the increment is elastic, it updates the stress according to the equations of elasticity; if the increment is elastic–plastic, it uses a NN to perform a regression similar as that in Model I.

Before proceeding to describe the details the two surrogate models, we define

4 $${{\varvec{\upvarepsilon}}}_{n} = \left( {\begin{array}{*{20}l} {\varepsilon_{z} } \\ {\varepsilon_{r} = \varepsilon_{\theta } } \\ {\varepsilon_{\theta z} } \\ \end{array} } \right);\quad {{\varvec{\upsigma}}}_{n} = \left( {\begin{array}{*{20}l} {\sigma_{z} } \\ {\sigma_{r} = \sigma_{\theta } = 0} \\ \tau \\ \end{array} } \right);\quad \Delta {{\varvec{\upvarepsilon}}}_{n} = {{\varvec{\upvarepsilon}}}_{n + 1} - {{\varvec{\upvarepsilon}}}_{n} ;\quad \Delta {{\varvec{\upsigma}}}_{n} = {{\varvec{\upsigma}}}_{n + 1} - {{\varvec{\upsigma}}}_{n} ;$$

as the current strain vector, current stress vector, strain increment vector, and stress increment vector, respectively; here *n* is an index denoting the current state while *n* + 1 denotes the state at the end of the simulation increment considered.

Considering the relatively small deformations applied to the specimens, we assume that additive strain decomposition applies, i.e.

5 $${{\varvec{\upvarepsilon}}} = {{\varvec{\upvarepsilon}}}^{el} + {{\varvec{\upvarepsilon}}}^{pl} = {\mathbf{S}}{\varvec{\upsigma }} + {{\varvec{\upvarepsilon}}}^{pl} .$$

We shall also make use of the current accumulated von Mises plastic strain, defined as

6 $$\begin{aligned} \overline{\varepsilon }_{n}^{pl} & = \sum\limits_{i = 1}^{n} \Delta \overline{\varepsilon }_{i}^{pl} = \sum\limits_{i = 1}^{n} {\sqrt {\frac{2}{3}\Delta {{\varvec{\upvarepsilon}}}_{i}^{pl} \Delta {{\varvec{\upvarepsilon}}}_{i}^{pl} } } \\ & = \sum\limits_{i = 1}^{n} {\sqrt {\frac{2}{3}\left[ {\left( {\Delta \varepsilon_{z}^{pl} } \right)_{i}^{2} + 8\left( {\Delta \varepsilon_{\theta z}^{pl} } \right)_{i}^{2} + 2\left( {\Delta \varepsilon_{r}^{pl} } \right)_{i}^{2} } \right]} } . \\ \end{aligned}$$

#### Model I

This model performs, at every increment, the regression.

7 $$\Delta {{\varvec{\upsigma}}}_{n} = NN_{R} \left( {\Delta {{\varvec{\upvarepsilon}}}_{n} ,\;{{\varvec{\upvarepsilon}}}_{n} ,\;{{\varvec{\upsigma}}}_{n} ,\;\overline{\varepsilon }_{n}^{pl} } \right)$$

As the material studied is elastic–plastic, its response is expected to be history-dependent. In this model, ${{\varvec{\upvarepsilon}}}_{n} ,\;{{\varvec{\upsigma}}}_{n}$ represent the current state of the material while $\overline{\varepsilon }_{n}^{pl}$ quantifies the effects of the previous history of plastic deformation. After the regression (7), the increments in the plastic strain vector are calculated as

8 $$\Delta {{\varvec{\upvarepsilon}}}_{n}^{pl} = \Delta {{\varvec{\upvarepsilon}}}_{n}^{{}} - {\mathbf{S}}\Delta {{\varvec{\upsigma}}}_{n}^{{}}$$

and the new accumulated plastic strain is computed from Eq. (6). The hyperparameters of the network $NN_{R}$ were decided by a trial and error procedure. The final network had two hidden layers with 128 neurons each. All inputs and outputs were scaled by the minmax() function^25^, and ReLU^26^ was the chosen activation function for all hidden layers. The training dataset was split into two portions, equal to 10% and 90% of the total, which were randomly selected as testing and training data, respectively. The Mean Absolute Error (MAE^25^) was used as the loss function after a preliminary study. The training of the NN was performed by backpropagation using the Adam optimiser^27^, with a number of epochs set to 70,000, a batch size of 200 and a learning rate of 0.001.

#### Model II

In a general increment in an FE simulation, two distinct behaviours (elastic or elastic–plastic) can be activated at each material point. The elastic response is simple to predict from knowledge of the compliance matrix **S**, while predicting the non-linear and history-dependent elastic–plastic response represents the real challenge. In Model II, we separate the two types of predictions, in an attempt to improve the surrogate model and to reduce its computational cost.

As shown in Fig. 6, the first step consists of a classification exercise to distinguish elastic from elastic–plastic increments. Mathematically

9 $$Y = NN_{C} \left( {\Delta {{\varvec{\upvarepsilon}}}_{n} ,{{\varvec{\upvarepsilon}}}_{n} ,{{\varvec{\upsigma}}}_{n} } \right)$$

where *Y* is the flag defined in Eq. (3) ($Y = 1$ corresponding to an elastic–plastic increment and $Y = 0$ corresponding to an elastic increment). $NN_{C}$ comprises 2 hidden layers containing 64 neurons each. The inputs were scaled by the minmax() function; ReLU was the chosen activation function for the 2 hidden layers while sigmoid^26^ was used for the output layer. A special dataset was assembled for this classification exercise, by selecting an equal number of datapoints (increments) with $Y = 1$ and $Y = 0$; the ratio of training to testing datapoints was again set to 90/10. The Binary Cross-Entropy (BCE) was the chosen loss function, as in^11^. The training of the NN was performed by backpropagation using the Adam optimiser^27^, with 10,000 epochs, a batch size of 100 and a learning rate of 0.001. The analysis of the confusion matrix^25^ showed that the classification was accurate in 99.84% of the cases.

For increments classified as elastic ($Y = 0$), the stress vector was updated as

10 $$\Delta {{\varvec{\upsigma}}}_{n} = {\mathbf{S}}^{ - 1} \Delta {{\varvec{\upvarepsilon}}}_{n}^{{}} .$$

For elastic–plastic increments, $NN_{R}$ was used to predict the increment in stress vector (Eq. (7)); the plastic strain increments were calculated according to Eq. (8), which allowed updating the accumulated plastic strain (Eq. (6)). In the case of Model II, $NN_{R}$ was trained using all datapoints with $Y = 1$; with the exception of the batch size, now set to 100 due to the reduced size of the dataset, all other parameters were identical to those reported above for Model I.

### Assessment of the accuracy of the surrogate models

**Figure 7:**
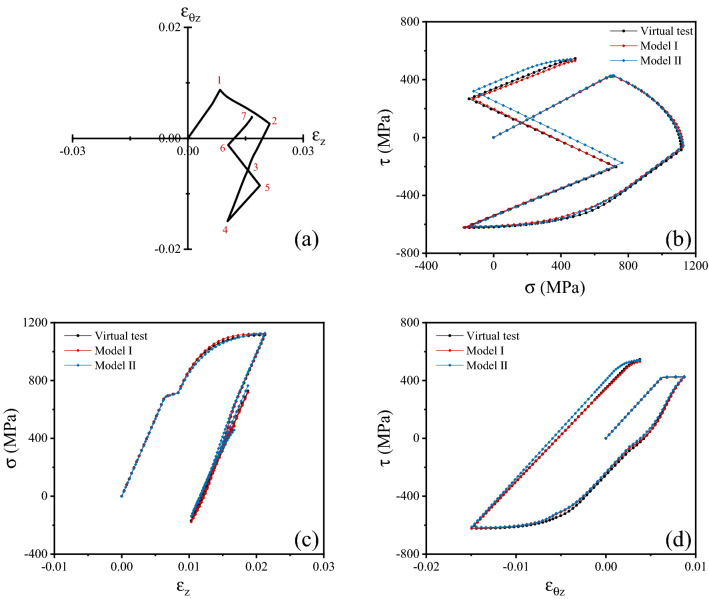
(**a**) One of the random strain histories used to assess the surrogate models; (**b**) stress history corresponding to the strain history in (**a**), including the predictions of the surrogate models; (**c**) normal stress versus strain history and predictions of the surrogate models; (**d**) shear stress versus strain history and predictions of the surrogate models.

**Figure 8:**
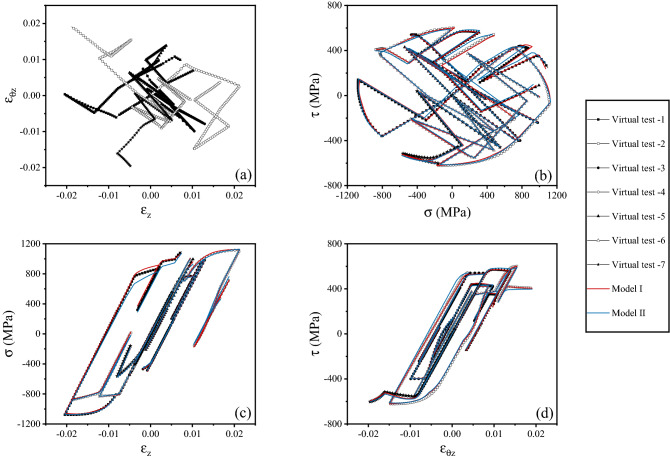
(**a**) Strain histories corresponding to seven random simulations used to assess the surrogate models; (**b**) stress history corresponding to the strain history in (**a**), including the predictions of the surrogate models; (**c**) normal stress versus strain history and predictions of the surrogate models; (**d**) shear stress versus strain history and predictions of the surrogate models.

To test the fidelity of the surrogate models developed above, we proceeded as follows. Twenty additional random strain histories were generated, and the corresponding virtual experiments were performed. This provided a set of datapoints (each corresponding to one increment from such simulations) which had not been seen by the surrogate models during their training. These were generated using the same procedure described in section “Generation of the training data set”. An example of such unseen strain histories and the corresponding stress histories are shown in Fig. 7, while Fig. 8 simultaneously presents 7 of such random strain histories.

Both Model I and Model II were used to predict the stress versus strain responses corresponding to the unseen random strain histories. For each of such histories, starting from the initial stress- and strain-free configuration (${{\varvec{\upvarepsilon}}}_{n = 1} = 0,\;{{\varvec{\upsigma}}}_{n = 1} = 0$), a sequence of strain increments $\Delta {{\varvec{\upvarepsilon}}}_{n}$, coinciding with those in the FE simulations (or virtual tests, representing here the ground truth), was provided as an input to the surrogate models; in each increment, the procedure in section “Surrogate models” was used to update the corresponding increments in stress vector and accumulated plastic strain. This allowed constructing predictions of the stress histories corresponding to each unseen random strain history.

## Results and discussion

The predictions of the surrogate models are compared to the ground truth (results of the virtual tests) in Figs. 7 and 8. Figure 7 presents the case of one selected random strain history; Fig. 8 shows the same information as in Fig. 7, but for the case of 7 selected random strain histories, to illustrate the accuracy of the models in wider regions of the strain and stress spaces.

We find that both surrogate models are in excellent agreement with the virtual tests. Both models capture the expected isotropy and symmetry of the material’s response, the yield locus corresponding to von Mises plasticity, and the details of the strain hardening characteristic of the solid. The accuracy of the surrogate predictions persists along most of the complex, non-monotonic, non-proportional strain paths considered; minor errors can be seen in the final parts of the random strain paths, due to accumulation of the error after a large number of increments, but also due to the relative scarcity of training datapoints for the NNs in those final parts of the random strain paths. We refer the reader to the Appendix (Supplementary material) for a brief discussion of the computational time required for the training and the use of the surrogate model.

The accuracy of the predictions reassures us that the proposed architecture of the surrogate models, including the history-dependent evolution of the accumulated plastic strain, is adequate to capture the material response of the isotropic von Mises solid considered in this study. We note that in^11^ we showed that similar architectures of the surrogate models were also adequate for fully dense solids displaying a pressure-sensitive response.

The obvious limitation of the surrogate models presented here that they are not necessarily accurate in regions of the input space scarcely populated by training datapoints. Such limitation can, however, be overcome by including additional coupon-level tests (real or virtual) to those considered here, for example biaxial or triaxial experiments. A complementary strategy could be to resort to physics-informed neural networks^28,29^: this would involve modifying the architecture of the surrogate models by enforcing appropriate constraints (for example: isotropy, symmetry and pressure-independence of the plastic response), including additional terms in the loss-function; it would however imply assuming a priori some features of the constitutive response. An additional development could be to include in the training dataset data measured in experiments involving non-uniform stress and strain fields, such as instrumented indentation or bending tests. We discuss this in the Appendix (Supplementary material).

## Conclusions

We proposed a methodology to develop data-driven surrogate constitutive models which do not rely on any constitutive theory and are informed exclusively from the results of carefully designed random non-proportional, non-monotonic multiaxial tests. The aim of this study is to develop accurate numerical tools to predict the elastic–plastic response of materials prior to establishing valid theories describing their behaviour, and without any knowledge of their microstructure.

We described a strategy to conduct relatively inexpensive experiments on the material of interest and to accurately capture its response by surrogate models based on small feed-forward NNs, including the design of suitable specimens and of the imposed random strain histories.

We demonstrated that the proposed framework is very effective in the specific case examined, and that the surrogate models presented capture all details of the constitutive response assigned to the specimens in the virtual experiments.

## Supplementary Information

Supplementary Information.

## Data Availability

The datasets used and analysed during the current study are available from the corresponding authors on reasonable request.
