# Supplementary material for: A strategy to formulate data-driven constitutive models from random multiaxial experiments
Source: Sci Rep. 2022 Dec 23;12:22248. doi: 10.1038/s41598-022-26051-y (PMC9789077; doi:10.1038/s41598-022-26051-y)
Supplement: Supplementary file 1 — Supplementary Information. [file 41598_2022_26051_MOESM1_ESM.docx]

**Supplementary material- Appendix**

**Dataset size and computational costs**

The accuracy of the predictions was found to increase with the size of the training dataset, in the range investigated in this study. To illustrate such sensitivity of the predictions to the dataset size Fig. A1 presents, for a selected test case, the evolution of the predictions when using datasets comprising 13800, 27600 and 55200 datapoints (i.e. 25%, 50% or 100% of the dataset produced). We progressively increased the training dataset size until the desired accuracy was achieved; we note however that even employing only 25% of the maximum number of training datapoints, the accuracy of the prediction would be more than adequate for a large range of applications. In this study the number of increments in the virtual tests was chosen to be the same as used by Abaqus. In real experiments more training datapoints could be extracted from each test, by just subdividing the measured histories of stress and strain in smaller or larger time increments.


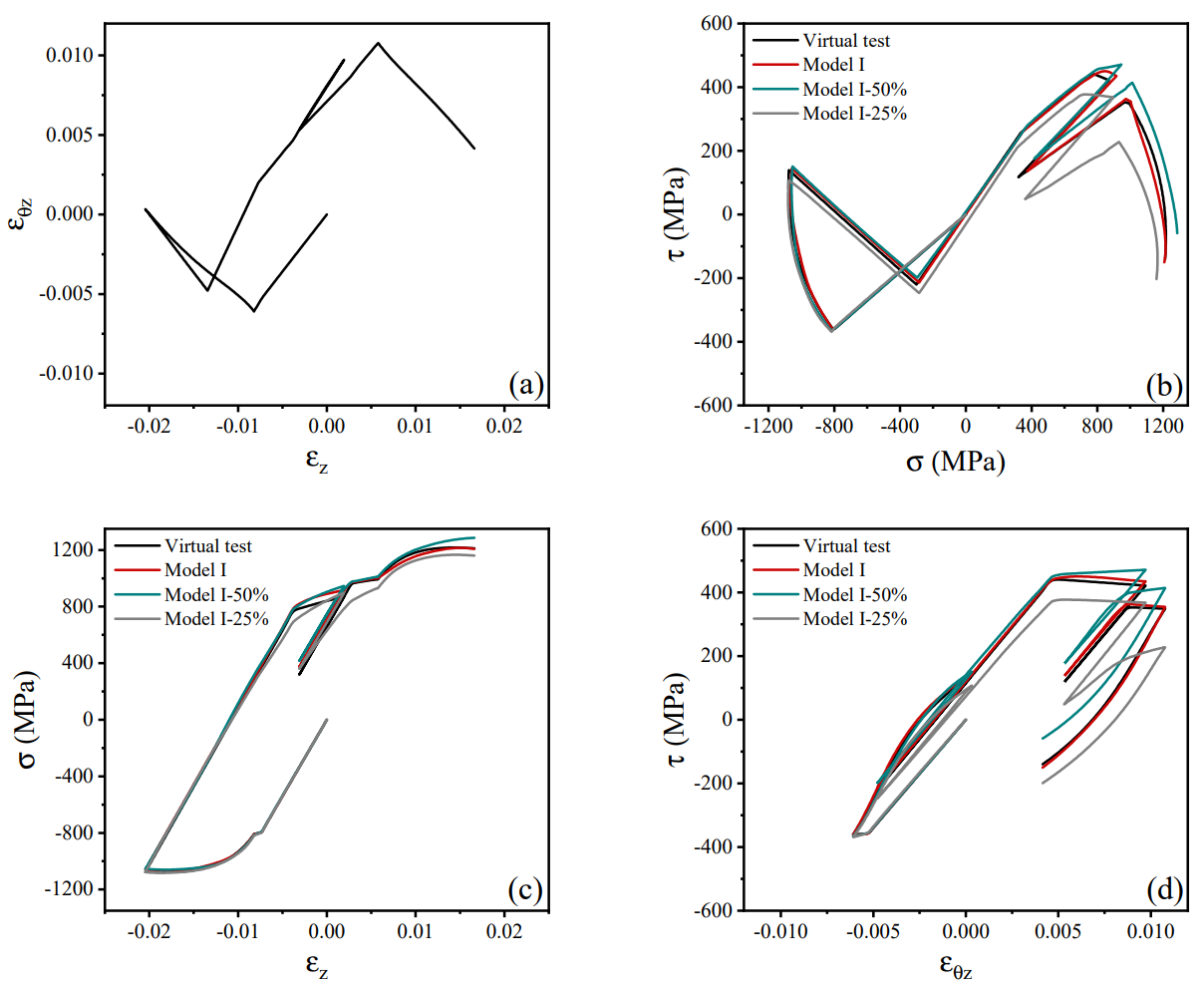


Figure A1. Predictions of model I with different datasets. (a) Selected random strain history. (b) Resulting stress history. (c) Normal stress vs strain history and predictions (d) shear stress vs strain history and predictions.

The training time of the NN was of approximately 7 hours using the complete training dataset (55200 increments) and an ordinary computer (2 x 2.6 GHz Intel Xeon CPU E5-2640 v3 processors, 64 GB of RAM). The time for training was approximately proportional to the size of the training dataset. With regards to the time required to perform the predictions, it is interesting to compare the performance of our surrogate model to that of the ordinary von Mises J2 isotropic plasticity model implemented in Abaqus. The ordinary model is slightly faster than the surrogate model in evaluating the stress update corresponding to a single prescribed increment in strain, as the surrogate model involves more complex algebraic operations. However, the evaluation of the stress increment corresponding to a given strain increment involves, when using the ordinary model, a number of iterations, while the surrogate model does not require this.

To quantify the relative speed of the ordinary and surrogate constitutive models, the surrogate model was implemented into a UMAT subroutine [A.1] suitable for beam elements. FE simulations were conducted considering a single beam element (element type B33 of Abaqus) fully constrained at one end and loaded by the application of combined axial displacement and torsional rotation at the opposite end. The beam had a thin-walled circular cross-section. The simulation time was set to 1 and the maximum time increment was set to 0.005. The simulation was run 2 times, describing the material’s behaviour with either the ordinary or the surrogate model. Both simulations completed with exactly 200 increments, each of duration 0.005, indicating that it was never necessary, in both cases, to reduce the time step. The simulation using the ordinary model was completed in 64 s, while that employing the surrogate model finished in 72 s. We note that in its current version, the UMAT subroutine performed one disk access at every evaluation, to read weights and biases of the NN; this step could be eliminated by employing a different coding structure. From this preliminary analysis we conclude that the surrogate model has similar computational cost as the analytical von Mises constitutive description.

**Extension of the surrogate models to 6-dimensional stress and strain spaces**

The surrogate models presented in this paper are expected to be accurate only in the input space defined by the training datapoints, in this case only for plane states of stress comprising one normal and one shear stress component. We note that, in numerous engineering applications, components are typically subject to deformation and loading within such range (e.g. loading of 2D beams and thin plates and shells). In other applications however the strain and stress tensors possess up to 6 non-negligible components. The neural networks can be generalised to provide predictions outside the domain of the training datapoints, but such predictions would extrapolate from the training data and likely they would not be accurate. The domain of applicability of the surrogate constitutive models can be extended following at least two strategies, outlined below.

The first strategy would involve making assumptions on the constitutive behaviour of the material; for example, for the isotropic elastic-plastic materials subject of this study, one could postulate a certain form of the functional dependence of the yield locus upon the stress and strain tensors and history variables, effectively adopting a chosen theoretical response. This approach however would require human judgement and it could be successful only in presence of a detailed understanding of the microstructure and of the active deformation mechanisms of the solid under consideration; as such, it would not be applicable for recently developed materials of unknown constitutive behaviour.

To obtain theory-free constitutive models it would be necessary to extend the ranges of input data spanned by the training datapoints. In practice, designing lab-scale experiments to span arbitrary six-dimensional states of stress and strain is not possible. In this study we focused on tension-torsion tests on thin-walled cylindrical specimens, as these are relatively easy to perform and allow obtaining approximately uniform states of strain and stress in the specimen, from which training datapoints can be directly obtained. The availability of biaxial and triaxial tests would somewhat extend the scope of the surrogate model, but not make it fully general; such tests (triaxial tests in particular) are also rare and costly. In contrast structural tests, in which the states of stress and strain are not uniform, are substantially easier to perform and it has been previously shown [A.2] that such tests can be used to train data-driven models.

Multiple structural tests could be designed to explore the stress and strain space as required. For example, non-monotonic random indentation tests would allow exploring states of (non-uniform) stress and strain with a high degree of hydrostatic compression; random combined bending/torsion tests on monolithic beams would allow further exploring 3D deformation and loading; tension/torsion tests on specimens of very low aspect ratio would allow obtaining data characterised by high hydrostatic tension; multiaxial loading of specimens and structures with large stress concentrators would interrogate the material’s response in presence of fully six-dimensional states of strain and stress.

In all the structural tests described above, the time histories of multiple displacements could be prescribed on the structure. Measurements would include the histories of the corresponding reaction forces, but an additional arbitrary number of surface strains and displacements could also be measured. A sufficiently rich set of lab-scale tests and structural tests could be used to train a surrogate model like that presented in this study, as follows: i) Discretise the set of loads, displacements and surface strain histories into small time increments, and split data in appropriate subsets for training, testing and validation. ii) Implement and initialise a general surrogate constitutive model, based on NNs with similar features as those described in this study (e.g a UMAT subroutine for Abaqus). iii) Conduct FE simulations of all the tests performed, describing the constitutive response of the material by the surrogate model. iv) Use a multi- or single-objective optimisation algorithm to determine the optimal weights and biases of the NN, based on the difference between measurements and FE predictions over each time increment. Appropriate loss functions could be devised to assign different relative importance to lab-scale tests (those resulting in uniform stress and strain histories), and the different measurements performed in the structural tests. Clearly, each evaluation of the loss function during the optimisation would require conducting multiple FE simulations, therefore the training times required would be substantially longer than those reported in this study, but we expect that these to be still within reach using relatively inexpensive hardware.

**References**

[A.1] Dassault Systemes. Abaqus/CAE User’s Guide 2017 (2016).

[A.2] Ghaboussi, J., Pecknold, D. A., Zhang, M. & Haj‐Ali, R. M. Autoprogressive training of neural network constitutive models. Int. J. Numer. Methods Eng. **42**(1), 105-126 (1998).
